# The development of empathy in the healthcare setting: a qualitative approach

**DOI:** 10.1186/s12909-022-03312-y

**Published:** 2022-04-04

**Authors:** Chou Chuen YU, Laurence TAN, Mai Khanh LE, Bernard TANG, Sok Ying LIAW, Tanya TIERNEY, Yun Ying HO, Beng Eng Evelyn LIM, Daphne LIM, Reuben NG, Siew Chin CHIA, James Alvin LOW

**Affiliations:** 1 Geriatric Education and Research Institute Ltd, Singapore, Singapore; 2 Department of Geriatric Medicine, Khoo Teck Puat Hospital, Singapore, Singapore; 3 Alice Lee Centre for Nursing Studies, Yong Loo Lin School of Medicine, National University of Singapore, Singapore, Singapore; 4 Lee Kong Chian School of Medicine, Nanyang Technological University, Singapore, Singapore; 5 Ministry of Health Holdings, Singapore, Singapore; 6 School of Health & Social Sciences, Nanyang Polytechnic, Singapore, Singapore; 7 Lee Kuan Yew School of Public Policy, National University of Singapore, Singapore, Singapore; 8 Tan Tock Seng Hospital, Singapore, Singapore

**Keywords:** Empathy, Empathy development, Empathy assessment, Empathy definition, Medical education

## Abstract

**Background:**

Healthcare professionals’ empathetic behaviors have been known to lead to higher satisfaction levels and produce better health outcomes for patients. However, empathy could decrease over time especially during training and clinical practice. This study explored factors that contributed to the development of empathy in the healthcare setting. Findings could be used to improve the effectiveness and sustainability of empathy training.

**Method:**

A qualitative approach, informed by aspects of grounded theory, was utilized to identify factors that enabled the development of empathy from the perspectives of doctors, nurses, allied healthcare workers and students. Twelve sessions of focus group discussions were conducted with 60 participants from two hospitals, a medical school, and a nursing school. Data was analyzed independently by three investigators who later corroborated to refine the codes, subthemes, and themes. Factors which influence the development of empathy were identified and categorized. This formed the basis of the creation of a tentative theory of empathy development for the healthcare setting.

**Results:**

The authors identified various personal (e.g. inherent characteristics, physiological and mental states, professional identity) and external (e.g. work environment, life experience, situational stressors) factors that affected the development of empathy. These could be further categorized into three groups based on the stability of their impact on the individuals’ empathy state, contributed by high, medium, or low stability factors. Findings suggest empathy is more trait-like and stable in nature but is also susceptible to fluctuation depending on the circumstances faced by healthcare professionals. Interventions targeting medium and low stability factors could potentially promote the development of empathy in the clinical setting.

**Conclusions:**

Understanding factors that impact the development of empathy allows us to develop measures that could be implemented during training or at the workplace leading to improve the quality of care and higher clinical work satisfaction.

## Introduction

Mercer and Reynolds [1] defined empathy in the medical context as the understanding of patients’ emotions, concerns and situations, communicating that understanding to the patient and acting on that understanding. Empathy improves diagnostic accuracy, patient satisfaction and compliance, and lowers psychological distress and medical complications [2–5]. Lack of empathy is correlated with physical, emotional, and work-related issues such as depression, burnout, sleep disturbance, and poor concentration, all of which could negatively impact patient care [6].

Despite extensive efforts to promote empathy through education, a decline in empathy has been observed among medical students, especially when they have spent more time interacting with patients [7–12]. This decline persists throughout residency and into their practice. Residents have been found to be less empathic and humanistic, and more cynical over time, while physicians from different specialties are at risk of compassion fatigue [7, 12–14]. While a decline in empathy was commonly reported in American medical schools, recent studies observed conflicting empathy trends in medical schools and empathy trends in other parts of the world were inconclusive [15, 16]. Consequently, this highlights a need to understand how clinical empathy develops among healthcare students and professionals.

Nezlek et al [17] believed that empathy should be considered both as a trait (a personal disposition that determines one’s ability to recognize, experience, and react to others’ emotions) and a state (the extent to which one empathizes with others in a specific event at a specific time). The same view was shared by Hojat [18] who considered empathy as neither a highly stable trait nor an easily fluctuating state, which was a result of a complex interplay of factors such as evolution, genetic dispositions, individual development, education and personal experiences. Hence, targeting these factors is thought to enable modification and development of empathy.

Many factors can affect an individual’s empathy level, such as gender, personality, career choices, common experience with patients, education background, and work environment. Females have been shown to have personality traits that lower stress levels [11, 19]. Medical students who prefer specialties with a more human touch [10, 11] have higher levels of empathy. Sharing common experiences with patients allowed healthcare professionals to empathize more with patients [20]. Medical education which focused more on science than humanities, and trainee distress are thought to lower empathy levels [7, 20–22]. Work experience and work environment could either positively or negatively influence empathy levels [23] while stress and burnout have been shown to lower empathy levels [5, 24].

Unfortunately, little is known about how these factors influence empathy at the trait and state levels. In social science, childhood experiences have been shown to have a long-lasting impact on individual trait empathy [5, 25]. On the other hand, cognitive load impedes empathy experience and reduces empathic responses [26], which is highly applicable to healthcare professionals as they constantly face massive workloads and responsibilities, thus affecting how they experience and exhibit empathy. While empathy research in healthcare has focused mainly on the experience of healthcare students and research on empathy interventions has focused solely on the success of these interventions, few have evaluated the development of empathy in healthcare workers [7, 8, 27–31]. Hence, the aim of this study is to qualitatively understand the underlying construct of empathy both as a trait and state in healthcare professionals and students, and determine what are the factors that may influence the development of empathy in the heatlhcare context.

## Method

### Research design

The research design was informed by the constructivist approach to grounded theory [32, 33] in which the aim was for researchers and participants to co-construct the theory on the development of empathy. A qualitative approach was adopted for this study as it was considered the most appropriate way to uncover and understand the meaning of empathy from the ‘emic’ perspective (i.e. the contexts, lives and meanings of those involved). This approach was also important considering that little is known about the theory of change whereby various factors influence the development of empathy of those experiencing empathy in the clinical setting. In view of practical constraints faced by the study team, approaches in grounded theory were adapted for the purpose of data collection and data analysis. Ethics approval for this study was granted by the National Health Group Domain Specific Review Board (DSRB), reference number 2018/00020.

### Data collection

Data was collected from care providers consisting of physicians, nurses, multidisciplinary teams, as well as medical and nursing students. While grounded theory would employ theoretical sampling to focus on and support a constant comparative analysis of data, this study adapted the sampling approach whereby clinicians on the study team made a strategic a priori decision based on their expertise to sample from various groups who would provide the most information-rich source of data. Healthcare professionals from various hospitals, medical students from a medical school and nursing students from a nursing school were invited via email to participate in the study. Participants were informed of the study details and written informed consent was obtained. Data was collected from 60 participants via 12 homogeneous focus group discussions (FGDs). Each FGD lasted approximately two hours. All FGDs were conducted in English and hence translation was not required. The demographic information is presented in Table 1.

**Table 1:** Demographic information of participants

	Profile of Participants					
	All	Doctor	Nurse	Multidisciplinary team	Medical student	Nursing student
Characteristics	*N* = 60	*N* = 5	*N* = 11	*N* = 5	*N* = 21	*N* = 18
Sex						
Female	40 (66.7)	5 (100)	10 (90.9)	3 (60)	7 (33.3)	15 (83.3)
Male	20 (33.3)	-	1 (9.1)	2 (40)	14 (66.7)	3 (16.7)
Race						
Chinese	39 (65)	4 (80)	5 (45.5)	4 (80)	18 (85.7)	8 (44.4)
Malay	9 (15)	-	1 (9.1)	1 (20)	1 (4.8)	6 (33.3)
Indian	5 (8.3)	-	1 (9.1)	-	2 (9.5)	2 (11.1)
Other	7 (11.7)	1 (20)	4 (36.4)	-	-	2 (11.1)
Education						
Secondary	2 (3.3)	-	1 (9.1)	-	-	1 (5.6)
Post-secondary (non-tertiary)	32 (53.3)	-	2 (18.2)	-	19 (90.5)	11 (61.1)
Polytechnic diploma	5 (8.3)	-	-	-	2 (9.5)	3 (16.7)
Bachelors	14 (23.3)	3 (60)	7 (63.6)	3 (60)	-	1 (5.6)
Masters	4 (6.7)	2 (40)	-	2 (40)	-	-
Professional Qualification	2 (3.3)	-	-	-	-	2 (11.1)
Postgraduate Diploma Certificate	1 (1.7)	-	1 (9.1)		-	-
Age (years)	M = 27.9 SD = 8.9	M = 32.6 SD = 3.9	M = 36 SD = 13.3	M = 33.4 SD = 7.9	M = 22.5 SD = 0.7	M = 26.4 SD = 7.4
Years of Practice		M = 6.4 SD = 5.8	M = 12 SD = 13.8	M = 9 SD = 8.8		

The FGDs were conducted in pairs by a female research officer (MK) with either a male medical doctor (LT), or male research fellow (CC) in rotation. All have practice experience in qualitative research and interviewing. MK and CC also had educational qualifications in psychology. Being a clinician, LT was able to reflexively use his knowledge of clinical practice to facilitate discussions in the clinical context whereas MK and CC approached the interviews from an outsider “naïve” position, thereby reducing the possibility of biasing the responses. For each FGD, one researcher would keep notes of the conversation to aid the interpretation of transcripts. Prior to the start of the FGD, each participant was provided with an information sheet containing details about the study and the research team introduced their roles in the study. Only researchers were present at all data collection settings except at the nursing school where the site investigators (part of the study team) were present to provide logistical support. These procedures in place adhered to common best practices to ensure trustworthiness in qualitative research [34]. Participants had no contact with the research team prior to study commencement.

The initial guided questions were broad and developed based on existing literature on empathy.

These questions focused on beliefs, thoughts, emotional feelings, behaviors and experiences and served as a guide to encourage participants to share their personal stories about their experiences of empathy especially in the clinical context and emerging themes were explored [33]. Examples of such questions included:

- What are your personal experiences of empathy in the care of patients?
- What are some of the things doctors or nurses do when they show empathy?
- Do you think empathy levels in someone can be changed? Or is it inborn, meaning it cannot be taught?
  Some questions were focused on more, or were included during subsequent interviews, as investigators felt that they were important issues that had surfaced during earlier interviews. This required the investigators to be sensitive and open to the views being shared. Examples of such questions included:
- Limited time to see patients is a factor that can influence empathy levels? What are your views on this?
- Some people are able to maintain their level of empathy despite personal or work related problems. Why do you think this is the case?
- Stressors at work can impact empathy levels. What is your view on this?

Negative case discussions were also encouraged as it allowed for emerging theories to be developed and modified while cases that did not fit led to generating of new knowledge [34]. Examples of such discussions included asking participants to discuss the negative consequences of having no empathy and possible negative effects that could result from having empathy. To ensure that the groups sampled were adequate, the investigators reviewed their field notes and logic diagram following each FGD to aid the assessment of saturation.

### Data analysis

Investigators met after each FGD to compare their memos, identify key themes generated by participants, compare findings with previous FGDs, and revise questions based on new themes that emerged. Upon completion of every two FGDs, the audio recordings were transcribed ad verbatim by one of two investigators and counterchecked against the recordings by LT. The investigators subsequently met regularly over a period of 12 months to compare codes and to form themes. Differences in opinion were mediated till a consensus was reached. This “immediate analysis” approach is an important part inspired by grounded theory [35] as it allowed the investigators to identify similarities and differences in the data. Additionally, it also informs the manner through which questions were developed and raised in each subsequent round of data collection.

Coding occurred in three stages [36]. First, open coding was conducted from the onset to generate as many ideas as possible regarding how empathy was described by participants, and whether the components of empathy could be categorized into the four domains postulated by the investigators. Axial coding then determined how the various codes related to each other throughout the dataset (e.g. factors related to childhood, environment, workplace, stress affecting empathy). Finally, selective coding involved the investigators selecting central core categories of ‘between and within person changes’ and ‘development over time’ and relating the codes to these categories. To support this whole process, diagrams of how the factors influenced participants’ empathy were constructed to identify the relationship between factors and categories after each FGD. A coherent theory of ‘empathy development’ was drafted by the 6^th^ FGD. This theory was further refined throughout the study until data saturation and this was achieved by the 12^th^ FGD.

## Results

**Fig. 1:**
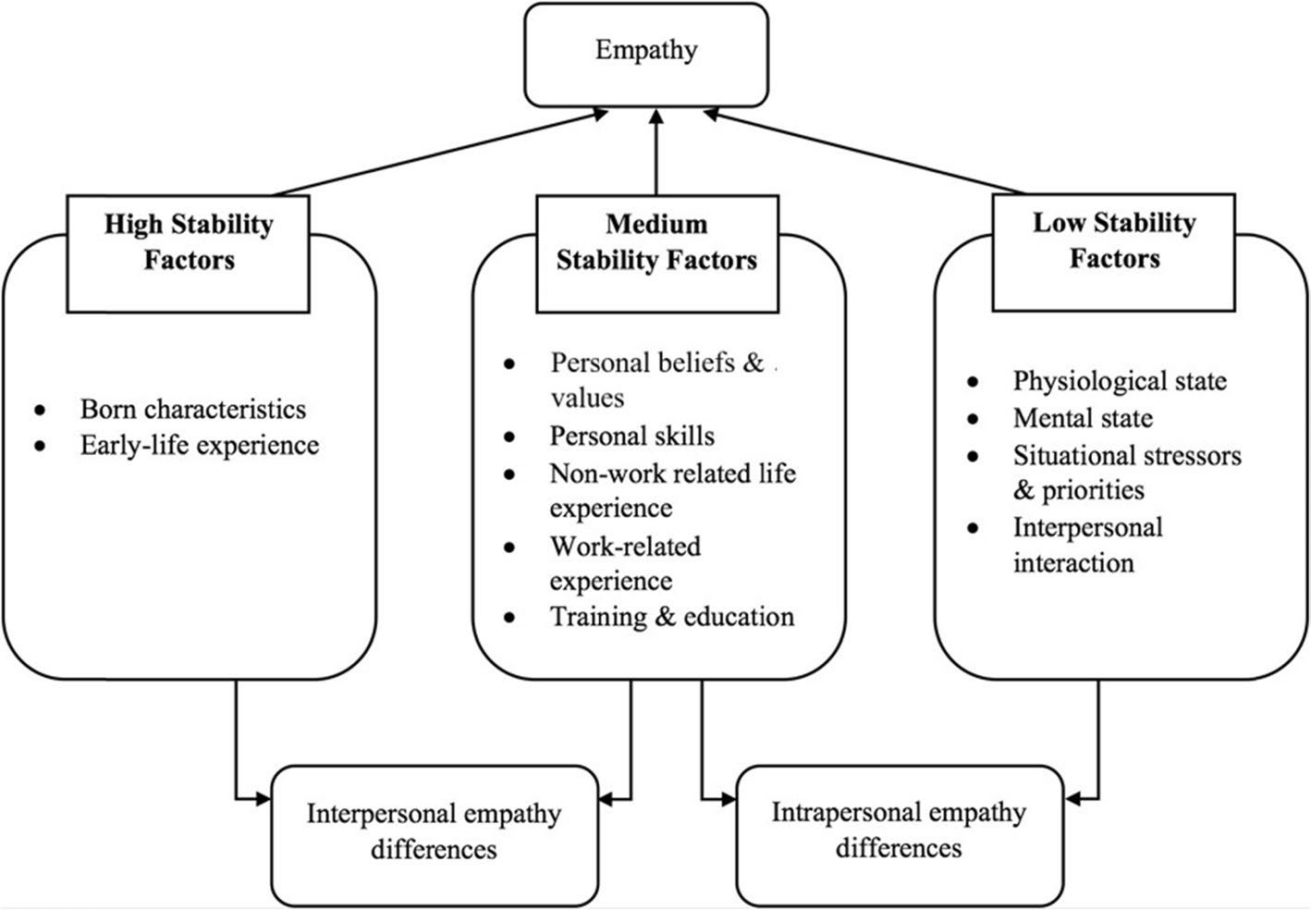
Empathy development model in healthcare setting

Participants on the whole considered empathy as both a trait and state. Although there were innate qualities that determined empathic tendencies and responses, these could also be learned and developed over time. Environmental and personal factors later in life are important determinants and these factors can be categorized according to difference in resistance to change which we termed ‘stability factors’ of which there are three levels: high, medium, and low. High stability factors form the foundation of an individual’s trait empathy (e.g. childhood experience, parental values and religious values). Their impact on one’s empathy, for instance, how one responses and reacts to others’ emotions, is long-lasting and less amenable to change. Low stability factors are those that are highly situation specific (e.g. unexpected stressors faced at work) whereby the impact is to cause momentary fluctuations in empathy levels. Medium stability factors are those that tend to be persistent and enduring in one’s environment (e.g. one’s job scope) and arguably have the potential to influence empathy levels over the long run. These factors often represent the environmental and personal constraints that exist for an extended period of time, and can influence one’s ability to empathize over the long run. The interaction between these factors and how they define empathy are presented in Fig. 1. All factors which influenced empathy, along with their representative quotes are listed in Table 2.

1. **High stability factors moulds trait empathy**

**Table 2:** Factors influencing empathy in healthcare settings

Impact level	Factors	Components	Quotes
High stability	Born characteristics	Personalities	“[…] everybody is born with a certain personality type. And whatever nurture you get beyond that is still working on the baseline that you’re already inborn with, and nurture might not be able to overcome what nature has already given you”. 20-FGD4
	Early life experience	Childhood experience	“I have a cousin who’s… he’s a child but he’s like quite- quite sick, always in and out of hospital, and he has a lot of medical problems, so I’ve seen how it affects like their family and then I always hear how like his mum, who’s my aunt, talk about a lot of things, you know, a lot of things they’re worried about, and things like that. So I guess sometimes when I speak to patients then I will be reminded of how sometimes all they want is to be reassured, or to know some things they’re really just worried about and they don’t know”. 22-FGD4
		Parental guidance	“So, little things, little experiences I still can remember, like people on the street asking for money and my mum will just put some in, and she said things like, ‘although this might not be much for us but to them it’s still a lot of money,’ and yeah just helping those in need, just doing little things through little experiences.” 16-FGD3
Medium stability	Non-work-related life experience		“I think actually another point really broadened my perspective was actually when we went for National Service. I think it really gave me a great deal of people from really diverse backgrounds that because previously the 12 formal years of education, it’s quite a small bubble, because we always hang around with people from very, very similar backgrounds. […] It helps you understand more like where people are coming from, or why people will approach certain problems in certain ways.” 12-FGD2
	Personal value and belief system	Sense of right and wrong	“I have certain personal biasness towards certain group of people. I know that’s my inherent biasness and it’s and professionally I shouldn’t have that kind of biasness, but I know I do. So these are the ones that I try a lot harder to work with. So one specific example is those with eating disorders, those anorexic. So to me, they do have an underlining issue. It may be psychiatric, it could be whatever. But it’s very difficult for me to empathize with them. Like, what makes you starve yourself?” 53-FGD11
		Professional identities	“If your top priority as a nurse, if let’s say, your own principle, your value as a nurse is number one to care for the patient, I think it will always be at the back of your mind. Even though you’re busy, you just find like ten seconds to just pop your head and tell the patient ‘are you okay, do you need my help?’ ‘No’ that’s all. Just merely ten seconds will do.” 48-FGD10
		Religion	“I mean some religions they teach about showing love and showing kindness to the people you meet, so definitely for some their religion would also teach them these values which would help them develop empathy.” 09-FGD2
		Culture & social norms	“Sometimes because of the culture that which you are from, maybe you're not aware that some of your behaviour hurt others. But- you don't have that, how to say, that- didn't (wasn’t) aware that some of your behaviour hurt others. But if someone tell you that your behaviour is not properly (proper) that's why (you) will hurt others, you better behaviour in the other way, if someone alert you, then you just realise then you will consciously (try) to change your behaviour.” 01-FGD1
	Personal skills	Communication skills	“[…] even though you get the translator, sometimes it might not be words to words translating you see, so you couldn't really get the meaning out of it, then you just guess a bit and all that.” 29-FGD6
		Emotion regulation & coping abilities	“Some people, like what I used to be along in my career, when it got too painful, I avoided. That was my strategy to deal with the feelings related to empathy that I couldn’t handle, at the earlier point of my life as a social worker.” 54-FGD11
	Work related experience	Work culture	“I think if you create an empathetic environment, I think generally anyone who works in that environment can actually pick up on it and they themselves be able to develop that soft skill as well, I feel, in a way. If your colleagues or people you’re working with do not display such attitude, then even if you yourself display such attitude, it may last you for a while. But perhaps after a long time, that might just die off as well.” 37-FGD7
		Work experience	“[…] after serving people through my career and things like that, the empathy kind of evolved into walking with you but not carrying you while I walk with you. The boundary making is a part of the thing now, I realized. Last time it used to be ‘woah I’m totally one with you’, like watching Korean drama, cry my eyes out and things like that. But now there’s a little bit of boundary, I know that I’m a watcher of the whole situation, I’m not in it and losing myself.” 54-FGD11
		Work guidelines & standard	“I would think also because structurally we have a lot of paper works that are legal documents. So we are very on task to fill up the documents more than talking to patient. Which is what happens the moment you graduate. Which is why staff who work long enough start to just become task oriented.” 60-FGD12
		Work set up	“[…] or even in terms of like the layout of the clinic room, for example, if like the doctor’s computer is facing the wall then end up like sometimes they may not even look at the patient, which makes the patient feel very neglected or like the doctor doesn’t care…” 13-FGD3
		Monitoring & reward system	“The kind of recognition that people get, I think, it’s very easy for organizations to recognize people who are very successful in terms of academic, very objective markers of success like academic or how many research papers you produce, how many patients you treat, what medal you won, but we don’t very often, we are doing it more now, but we don’t very often reward people for empathy obviously because it’s harder to see someone displaying empathy, because it’s a very one-to-one thing but I think recognizing it also tilts the focus towards that and makes people realize that it’s actually a more important aspect of the care that you give.” 17-FGD3
		Supervisor influence	“[…] different senior doctors have different preferences when it comes to presentations, so, in the same way, if they don’t consider showing empathy a very important…, or they think it’s just a waste of time, then definitely juniors who are still learning how to become doctors, they would adjust and they would learn accordingly.” 21-FGD4
		Role model	“[…] it’s also perhaps significant people along the way that affects us, that bolds us, that changes our way of thinking, our reaction or coach or mental guide. So I think that is also significant in shaping me as a healthcare professional.” 53-FGD11
	Training & education		“What’s being taught nowadays in school is not so much how to care, but how to show- or how to pretend that you’re caring. At the start you know we’re just pretending, you know, but after going through our clinical years you sort of understand what it means to care and how- what kind of impact it can have and I think that in itself is sort of teaching you like empathy in a sense that why you need to care. And the how to show you’re caring, it sort of makes sense once you see why you need to care.” 07-FGD2
Low stability factors	Physiological states		“[…] sometimes really you just didn’t have enough sleep, you didn’t each much, you just get really tired, you just don’t want to care.” 60-FGD12
	Mental states	Mood	“If I'm actually in a terrible mood in the morning and usually the first patient of the day usually gets it. (Laughter) I mean we'll try not to, cause we remind ourselves ‘you shouldn't vent’, but sometimes I mean you're all human right.” 31-FGD6
		Burnout	“Anyone who’s worn out definitely will try to protect themselves more. Like look at survival situations. Nobody’s going to care about other people, they only care about themselves.” 59-FGD12
	Situational stressors & priorities		“Usually in the most emergency situations also, empathy takes the backseat. I would say that the priority is to stabilise the patient. But of course at the same time you try to maintain dignity and all, you try to not let patient suffer”. 33-FGD6
	Interpersonal interactions	Patient-healthcare professional relationship	“I mean some of the family members are just rude and harsh and they expect us in a way like robots, emotionless. So it’s like whatever they can do, like they are hurt by the doctors diagnosis, results, wrong results, like results everything, so they will push the blame on the nurse. And, it kind of triggered us to the point where we will suddenly just burst and treat harshly to the patient, to the family members. And that will effect over (affect) empathy.” 47-FGD9

High stability factors were often the first thoughts that came to the participants’ mind when asked where they derived their sense of empathy from. Participants believed that individual baseline empathy determined their tendency to empathize with others and was shaped by inborn characteristics and early life experiences. The influence of high stability factors on empathy was persistent and fairly stable. Some participants felt that factors which occurred at the later stages of life, such as empathy training in schools, may not be able to fundamentally change an individual’s trait empathy level and response:

“[…] everybody is born with a certain personality type. And whatever nurture you get beyond that is still working on the baseline that you’re already inborn [born] with, and nurture might not be able to overcome what nature has already given you”. 20-FGD4.

As different individuals have different upbringing and early life experiences, high stability factors also seemed to explain the differences in empathy levels among individuals.

“I think it depends on the person’s upbringing and the environment they grew up in ‘cause [because] throughout my life I’ve seen a lot of people who are able to put themselves in other people’s shoes and some who just aren’t.” 59-FGD12.

2. **Medium stability factors can have enduring effects empathy levels**

Medium stability factors can have an important influence on empathy levels, despite less permanent impact than that of high stability factors. These include current belief and value systems, education and training, group influences, work experiences and culture, supervisory influences, and the professional identity that one adopts. In the clinical context, values in medical practice guides how one understands or appreciates another person’s behavior and situation, which in turn influences their empathy level and response. Additionally, factors such as emotion regulation, coping capabilities, perspective-taking, self-reflective ability, as well as verbal and non-verbal communication skills, allowed healthcare professionals to feel, understand, and communicate better in response to patients’ emotions and reactions.

Participants also shared that religious teachings or other forms of educational training could benefit in helping one empathize with others. Tools and frameworks from educational training helped in relating and communicating affectively, for instance, what to say and do in certain scenarios faced by the patients. Interestingly, with more life experiences, healthcare professionals found it easier to relate to the lived experiences of patients. Work experiences could also improve emotional maturity, knowledge acquisition, coping strategies and communication skills:

“… empathy also comes from your experience, as all of them have mentioned earlier, like the kind of experiences you’ve been through which allows you to put yourself in the shoes of these patients which you’ll be seeing, and also having that experience of [for example] like say breaking bad news to this patient multiple times, I would learn how to do it better, and improve myself like maybe the fifth [time] and by- compared to the like one hundredth time I’ve done it, so I think if I had to choose one I think empathy is something that yes, as- there’s a basal level of like inborn like empathy, but it can definitely be developed and honed, so that you are able to like connect with your patients better.” 25-FGD5.

Most participants felt that it was easier to empathize with patients when they had more experience. Participants also shared various examples of how work responsibilities, standards and guidelines, culture, supervisors, surveillance, and reward structure affected empathy levels, as shown in Table 2. Despite the stress and various challenges that came with the role of providing care, participants highlighted that their professional identities spurred them to maintain empathy even in difficult times, and they did this by contemplating what it meant to be a healthcare professional as well as prioritizing work and responsibilities of patient care.

Although participants felt that the impact of high stability factors was harder to alter, empathy levels could still change over time through interactions with medium stability factors. In some situations, they could override the influence of high stability factors, as mentioned by one participant:

“Parents teach us [to] always be nice to people, do things nicely. But when we step out to the world, we see like the world is not actually friendly. We try to be nice to people but they just shut us out. So it’s a different kind of empathy, and what triggers this empathy in us is experiences. How we see things, how we mature ourselves and for example if we have a situation at hand, how we handle it, how we show our feelings, is different from what our parents would teach us. And depends, either we ourselves would want to follow what our parents have taught, or we want to change and adapt to it.” 47-FGD9.

Similar to high stability factors, there are inter-individual differences in medium stability factors. At the same time, these factors of influence do change over time (e.g. transitioning from medical school to the clinical setting resulted in changes in work responsibilities and expectations). Hence, medium stability factors could account for both interpersonal (between individuals) and intrapersonal (within an individual at different time points) differences in empathy level.

3. **State empathy fluctuates due to low stability factors**

While trait empathy is relatively stable, one could still experience transient fluctuations in the experience and expression of empathy due to the presence of low stability factors. These factors often acted as short-term barriers or facilitators that determined how healthcare professionals felt or expressed empathy in a given situation:

“… how much innate empathy you have and then your experiences, and then how that leads to how much empathy you feel, but how much you express depends also a lot on the circumstances of the practice, and how much time you have.” 28-FGD5.

Low stability factors in individuals ranged from physiological states, such as being tired or hungry, to psychological conditions, such as stress and anxiety. Occasionally, situational imperatives and demands such as in the case of a medical emergency, made it challenging to feel or express empathy. Negative interactions and poor rapport between healthcare professionals and patients or family members (e.g. rude demands from family members) that affected mood could also hamper empathic response whereas positive interactions have been reported to have the opposite effect.

With prolonged negative interactions, participants shared that healthcare professionals in certain conditions might become ‘desensitized’ or even experience burnout, leading to avoidance behavior as a coping strategy, with negative consequences on empathy levels. However, high and medium stability factor can have protective effects and buffer against some of the negative interactions. As one participant shared in response to negative experiences faced by healthcare professionals, possessing strong trait empathy and a supportive work environment might protect individuals from the effects of stress or burnout, and help individuals maintain their empathy level:

“It’s tough. I mean if you got it you got it. May [Maybe] you got a very strong empathy, I think you can keep that. […] Maybe situation supports them; maybe the environment supports them to be there.” 38-FGD7.

## Discussion

Understanding empathy in the clinical setting allows trainers and mentors to focus on factors which will positively influence empathy development in clinicians. Our study showed concurring opinions among students and healthcare professionals with regard to empathy development in clinicians. The key findings suggest that the factors affecting empathy development can be categorized into high, medium, and low stability factors, which explains the inter-individual and intra-individual variations in the experiences and expressions of empathy.

In line with past research, high stability factors such as inborn personal characteristics have been shown to influence empathy [11, 37, 38]. This is also the case for other factors identified such as childhood influence from family members and parenting style [39, 40]. As highlighted by the participants, social interactions during childhood including school experiences could also determine an individual’s emotional and prosocial tendency development [5, 25].

Medium stability factors were also found to be important in the development of empathy. As with prior research, the findings suggest that empathy development could be influenced by whether one’s values prioritizes the welfare of others [41]. The relationship between religion and empathy was another area of interest among researchers. In line with what other studies have found, while religion seemed to have an influence on prosocial behaviors, the relationship between religion and empathy was affected by how individuals interpreted religious teachings [42, 43]. Similarly, culture was also found to have an influence on empathy [44]. As culture often dictates communication norms, this determines the ability to build trust between patients and healthcare professionals as well as the perception of empathy in healthcare settings [45].

Factors attributable to the erosion or development of empathy during medical school training and clinical practice which were reported in previous studies were also found in our study. The inability to relate to patients due to lack of life experience or contact with patients, negative encounters with patients, heavy workload, desensitization, burnout, stress, hostile work environment, training, and work culture could lower empathy level. On the other hand, emphasizing the value of empathy during training or in the work culture, interactions with a role model and supervisor, and conducting communication training could improve empathy [46–57].

Our findings on the effects of low stability factors generally mirrored previous studies where mental state, situational stressors, and interpersonal interactions were found to influence empathy [7, 24, 48, 49]. Similar to an exploratory study by Pohontsch et al. [50], we found that negative mood, work stress, lack of time, and negative interactions with patients, inhibited empathy although our study included not only students but also healthcare professionals. Other than one study in the healthcare setting that the authors are aware of, extant evidence is limited regarding the effects of physiological state (e.g. mood, hunger, fatigue) on empathy and findings from this study add to the literature by suggesting they can have detrimental effects. Thomas et al. [48] showed that well-being correlated positively with empathy whilst poor sleep impacted the capacity of mental health nurses to provide empathic and compassionate care [58]. Such effects on empathy were also reported in our sample. Supporting the well-being of trainees and clinicians, as well as investing in a healthy work-place culture that includes measures to protect healthcare professionals from verbal abuse, could therefore be important.

Overall, findings from this sample suggest that empathy tended to be more trait-like and stable in nature but is also susceptible to regular fluctuation depending on the circumstances healthcare professionals find themselves in. The stability of their effect has been studied mostly in the field of social and developmental psychology. The work of Knafo and colleagues [59] demonstrated the influence of genetic and environmental factors on empathy development at an early age. Empathy was found to be a stable disposition determined by genetics but could change due to both genetic and environmental factors. The environmental variables shared by children could explain empathy stability while non-shared environmental variables determined the change in empathy. Taylor et al. [60] showed that the long-lasting impact of personalities, parental guidance, and experiences on empathy at an early age were able to predict future prosocial behaviors. In addition, Greenberg et al. [61] showed that people who experienced traumatic events when they were young tended to have higher levels of empathy at adulthood.

### Implications for practice

Our tentative theory of empathy development provides a framework to understand potential targets for empathy interventions. While targeting high stability factors may not be possible in an attempt to change trait empathy, developing the manner healthcare professionals/students understand, relate and respond emphatically to patients in medical or nursing schools as well as other clinical settings can be achieved and sustained by targeting both medium and low stability factors. Attempts to improve empathy in medical schools, nursing schools, and clinical practice over the years have largely been focused on social skills and perspective-taking [30, 31, 62, 63]. A recent longitudinal study of Japanese medical students showed that communication skills education could improve empathy, but the effect was short-lived [64]. The challenge with focusing on social skills alone was that it often felt forced into a teaching curriculum as individuals were not always able to feel authentic empathy in simulated settings [46]. Shapiro et al. [65] was more successful in creating a sustainable positive effect by targeting different factors such as communication skills, coping techniques, well-being enhancing strategies, and exposure to patients; these are some of the factors outlined in our proposed model which adopted a more experiential approach in a real-life setting.

Our theory of empathy development is holistic and highlights that healthcare professionals should be equipped with the necessary skills, experience, and guidance to react empathically in the clinical setting, and that their work environment has to be conducive to minimize the effect of low stability factors. For example, forming healthcare students’ and professionals’ professional identity at an early stage and regularly reinforcing the identity, creating a supportive work culture, training and education, supervisory guidance and peer influence, and even developing a monitoring system that rewards empathic behaviors could help eliminate the effect of low stability factors on empathy.

### Strengths and limitations

The strength of this study involves understanding views from a sample of doctors, nurses, multidisciplinary team members, medical students and nursing students and findings is therefore not narrowly confined to only one group, which is quite typical for qualitative research. In addition, this study was conducted in a multi-cultural setting with participants from different ethnic groups, religious beliefs and work setting (acute hospital, community hospital, home care and schools). With findings echoing those found by scholars in the field of empathy, this suggests that the theory of empathy development is applicable in the international community as it provides a framework to understand potential targets for empathy interventions.

One limitation was that as mentioned in the method section, theoretical sampling was not used. This would be expected for any study that adhered strictly to grounded theory. The sampling procedure therefore was guided by strategic a priori decision based on the expertise of the clinicians in the study teams that was in part guided by situational constraints and access especially with regards to the doctors and nurses. As there were more participants coming from ‘high-touch’ clinical setting such as palliative care and geriatrics, future studies may need to consider whether views about empathy from other settings such as the emergency department may differ. As identified in this study, participants felt that empathy levels may be affected in highly demanding clinical situations such as an emergency.

Another limitation of this study is that the use of FGDs may have induced socially desirable responses from participants. For the healthcare professionals, there is a possibility that what was shared may not be truly reflective of their personal views since the sessions were conducted in the presence of fellow colleagues from the same institution. Likewise, the study team also felt that the role of religion was not fully explored in the FGDs as there were instances participants did not feel comfortable or appropriate sharing their personal views on religion in the presence of other fellow medical professionals and associates.

## Conclusion

To a large extent, empathy is an inborn trait and fundamental to being human. However, it is dynamic, constantly evolving, and develops under the influence of various personal and situational factors. Our proposed theory of empathy development consolidates the factors influencing empathy and describes their involvement in influencing empathy over time both intra-personally and inter-personally. With a clearer understanding of how empathy develops in the healthcare setting, quality of clinical care in the future may be improved as healthcare providers could implement measures during training or at the workplace, to encourage empathy and compassion in healthcare.

## Data Availability

The authors declare that the data supporting the findings of this study are available within the article.

## Abbreviation

FGD: Focus group discussion

## References

[1] Mercer SW, Reynolds WJ (2002). Empathy and quality of care. Br J Gen Pract..

[2] Rakel DP, Hoeft TJ, Barrett BP, Chewning BA, Craig BM, Niu M (2009). Practitioner empathy and the duration of the common cold. Fam Med.

[3] Derksen F, Bensing J, Lagro-Janssen A (2013). Effectiveness of empathy in general practice: a systematic review. Br J Gen Prac.

[4] Larson EB, Yao X (2005). Clinical empathy as emotional labor in the patient-physician relationship. JAMA.

[5] Neumann M, Bensing J, Mercer S, Ernstmann N, Ommen O, Pfaff H. Analyzing the “nature” and “specific effectiveness” of clinical empathy: a theoretical overview and contribution towards a theory-based research agenda. Patient Educ Couns. 2009;74(3):339–46. 10.1016/j.pec.2008.11.013. 10.1016/j.pec.2008.11.013 19124216

[6] Sorenson C, Bolick B, Wright K, Hamilton R (2016). Understanding compassion fatigue in healthcare providers: a review of current literature. J Nurs Scholarsh.

[7] Neumann M, Edelhäuser F, Tauschel D (2011). Empathy decline and its reasons: a systematic review of studies with medical students and residents. Acad Med.

[8] Pedersen R (2010). Empathy development in medical education–a critical review. Med Teach.

[9] Stratton TD, Saunders JA, Elam CL. Changes in medical students’ emotional intelligence: an exploratory study. Teach Learn Med. 2008;20(3):279–84. 10.1080/10401330802199625. 10.1080/10401330802199625 18615305

[10] Hojat M, Vergare MJ, Maxwell K (2009). The devil is in the third year: a longitudinal study of erosion of empathy in medical school. Acad Med.

[11] Chen D, Lew R, Hershman W, Orlander J (2007). A cross-sectional measurement of medical student empathy. J Gen Intern Med.

[12] Gribben JL, Kase SM, Waldman ED, Weintraub AS (2019). A cross-sectional analysis of compassion fatigue, burnout, and compassion satisfaction in pediatric critical care physicians in the United States. Pediatr Crit Care Med.

[13] Collier VU, McCue JD, Markus A, Smith L (2002). Stress in medical residency: status quo after a decade of reform?. Ann Intern Med.

[14] Hunt P, Denieffe S, Gooney M (2019). Running on empathy: Relationship of empathy to compassion satisfaction and compassion fatigue in cancer healthcare professionals. Eur J Cancer Care (Engl).

[15] Andersen FA, Johansen AB, Søndergaard J, Andersen CM, Assing HE. Revisiting the trajectory of medical students’ empathy, and impact of gender, specialty preferences and nationality: a systematic review. BMC Med Educ. 2020;20(1):52. 10.1186/s12909-020-1964-5. 10.1186/s12909-020-1964-5 PMC7027232 32066430

[16] Ferreira-Valente A, Monteiro JS, Barbosa RM, Salgueira A, Costa P, Costa MJ (2017). Clarifying changes in student empathy throughout medical school: a scoping review. Adv Health Sci Educ Theory Pract.

[17] Nezlek JB, Schütz A, Lopes P, Smith CV. Naturally occurring variability in state empathy. Empathy in Mental Illness. 2007;187–200. 10.1017/cbo9780511543753.012

[18] Hojat M (2007). Empathy in Patient Care: Antecedents, Development, Measurement, and Outcomes.

[19] McManus IC, Keeling A, Paice E. Stress, burnout and doctors’ attitudes to work are determined by personality and learning style: a twelve-year longitudinal study of UK medical graduates. BMC Med. 2004;2:29. 10.1186/1741-7015-2-29. 10.1186/1741-7015-2-29 PMC516448 15317650

[20] Ekman E, Krasner M (2017). Empathy in medicine: Neuroscience, education and challenges. Med Teach.

[21] Jeffrey D. A meta-ethnography of interview-based qualitative research studies on medical students’ views and experiences of empathy. Med Teach. 2016;38(12):1214–20. 10.1080/0142159X.2016.1210110. 10.1080/0142159X.2016.1210110 27552411

[22] Eikeland HL, Ørnes K, Finset A, Pedersen R. The physician’s role and empathy - a qualitative study of third year medical students. BMC Med Educ. 2014;14:165. 10.1186/1472-6920-14-165. 10.1186/1472-6920-14-165 PMC4128827 25108627

[23] Gleichgerrcht E, Decety J (2014). The relationship between different facets of empathy, pain perception and compassion fatigue among physicians. Front Behav Neurosci.

[24] Wilkinson H, Whittington R, Perry L, Eames C (2017). Examining the relationship between burnout and empathy in healthcare professionals: A systematic review. Burn Res.

[25] Volling BL, Kolak AM, Kennedy DE. Empathy and Compassionate Love in Early Childhood: Development and Family Influence. The science of compassionate love: Theory, research, and applications. 2008; 161–200 10.1002/9781444303070.ch6

[26] Meiring L, Subramoney S, Thomas KG, Decety J, Fourie MM (2014). Empathy and helping: effects of racial group membership and cognitive load. South African Journal of Psychology.

[27] Pedersen R (2009). Empirical research on empathy in medicine—a critical review. Patient Educ Couns.

[28] Sulzer SH, Feinstein NW, Wendland CL (2016). Assessing empathy development in medical education: a systematic review. Med Educ.

[29] Yu J, Kirk M (2008). Measurement of empathy in nursing research: systematic review. J Adv Nurs.

[30] Kelm Z, Womer J, Walter JK, Feudtner C (2014). Interventions to cultivate physician empathy: a systematic review. BMC Med Educ.

[31] Batt-Rawden SA, Chisolm MS, Anton B, Flickinger TE (2013). Teaching empathy to medical students: an updated, systematic review. Acad Med.

[32] Charmaz K (2004). Premises, principles, and practices in qualitative research: Revisiting the foundations. Qual Health Res.

[33] Charmaz K. Constructing grounded theory: A practical guide through qualitative analysis. Sage Publicatoins; 2006 Jan 13.

[34] Lincoln YS, Guba EG. Naturalistic inquiry. Sage Publications; 1985.

[35] Engward H. Understanding grounded theory. Nursing Standard (through 2013). 2013 Oct 16;28(7):37. 10.7748/ns2013.10.28.7.37.e7806 24128248

[36] Strauss A, Corbin J. Basics of qualitative research. Sage Publications; 1990.

[37] Austin EJ, Evans P, Magnus B, O’Hanlon K. A preliminary study of empathy, emotional intelligence and examination performance in MBChB students. Med Educ. 2007;41(7):684–9. 10.1111/j.1365-2923.2007.02795.x. 10.1111/j.1365-2923.2007.02795.x 17614889

[38] Newton BW, Barber L, Clardy J, Cleveland E, O’Sullivan P. Is there hardening of the heart during medical school? Acad Med. 2008;83(3):244–9. 10.1097/ACM.0b013e3181637837. 10.1097/ACM.0b013e3181637837 18316868

[39] Strayer J, Roberts W (1989). Children’s empathy and role taking: Child and parental factors, and relations to prosocial behavior. J Appl Dev Psychol.

[40] Yoo H, Feng X, Day RD. Adolescents’ empathy and prosocial behavior in the family context: a longitudinal study. J Youth Adolesc. 2013;42(12):1858–72. 10.1007/s10964-012-9900-6. 10.1007/s10964-012-9900-6 23283695

[41] Balliet D, Joireman J, Daniels D, George-Falvy J (2008). Empathy and the Schwartz value system: a test of an integrated hypothesis. Individ Differ Res.

[42] Saroglou V, Pichon I, Trompette L, Verschueren M, Dernelle R (2005). Prosocial behavior and religion: new evidence based on projective measures and peer ratings. J Sci Study Relig.

[43] Duriez B (2004). Are religious people nicer people? Taking a closer look at the religion–empathy relationship. Ment Health Relig Cult.

[44] Chung W, Chan S, Cassels TG (2010). The role of culture in affective empathy: cultural and bicultural differences. J Cogn Cult.

[45] Lorié Á, Reinero DA, Phillips M, Zhang L, Riess H (2017). Culture and nonverbal expressions of empathy in clinical settings: A systematic review. Patient Educ Couns.

[46] Laughey WF, Brown MEL, Finn GM. 'I'm sorry to hear that'-Empathy and Empathic Dissonance: the Perspectives of PA Students. Med Sci Educ. 2020; 1–10. doi:10.1007/s40670-020-00979-0 10.1007/s40670-020-00979-0 PMC7224074 32421094

[47] West CP, Shanafelt TD (2007). The influence of personal and environmental factors on professionalism in medical education. BMC Med Educ.

[48] Thomas MR, Dyrbye LN, Huntington JL (2007). How do distress and well-being relate to medical student empathy? a multicenter study. J Gen Intern Med.

[49] Bayne H, Neukrug E, Hays D, Britton B (2013). A comprehensive model for optimizing empathy in person-centered care. Patient Educ Couns.

[50] Pohontsch NJ, Stark A, Ehrhardt M, Kötter T, Scherer M (2018). Influences on students’ empathy in medical education: an exploratory interview study with medical students in their third and last year. BMC Med Educ.

[51] Winseman J, Malik A, Morison J, Balkoski V. Students’ views on factors affecting empathy in medical education. Acad Psychiatry. 2009;33(6):484–91. 10.1176/appi.ap.33.6.484. 10.1176/appi.ap.33.6.484 19933894

[52] Bandini J, Mitchell C, Epstein-Peterson ZD (2017). Student and faculty reflections of the hidden curriculum. Am J Hosp Palliat Care.

[53] Meadors P, Lamson A (2008). Compassion fatigue and secondary traumatization: provider self care on intensive care units for children. J Pediatr Health Care.

[54] Smith-MacDonald L, Venturato L, Hunter P (2019). Perspectives and experiences of compassion in long-term care facilities within Canada: a qualitative study of patients, family members and health care providers. BMC Geriatr.

[55] Dev V, Fernando AT, Kirby JN, Consedine NS (2019). Variation in the barriers to compassion across healthcare training and disciplines: A cross-sectional study of doctors, nurses, and medical students. Int J Nurs Stud.

[56] Epstein RM, Hadee T, Carroll J, Meldrum SC, Lardner J, Shields CG. “Could this be something serious?” reassurance, uncertainty, and empathy in response to patients’ expressions of worry. J Gen Intern Med. 2007;22(12):1731–9. 10.1007/s11606-007-0416-9. 10.1007/s11606-007-0416-9 PMC2219845 17972141

[57] Vogel D, Meyer M, Harendza S (2018). Verbal and non-verbal communication skills including empathy during history taking of undergraduate medical students. BMC Med Educ.

[58] Gerace A, Rigney G (2020). Considering the relationship between sleep and empathy and compassion in mental health nurses: It’s time. Int J Ment Health Nurs.

[59] Knafo A, Zahn-Waxler C, Van Hulle C, Robinson JL, Rhee SH (2008). The developmental origins of a disposition toward empathy: genetic and environmental contributions. Emotion.

[60] Taylor ZE, Eisenberg N, Spinrad TL, Eggum ND, Sulik MJ (2013). The relations of ego-resiliency and emotion socialization to the development of empathy and prosocial behavior across early childhood. Emotion.

[61] Greenberg DM, Baron-Cohen S, Rosenberg N, Fonagy P, Rentfrow PJ (2018). Elevated empathy in adults following childhood trauma. PLoS ONE.

[62] Levett-Jones T, Cant R, Lapkin S (2019). A systematic review of the effectiveness of empathy education for undergraduate nursing students. Nurse Educ Today.

[63] Bas-Sarmiento P, Fernández-Gutiérrez M, Baena-Baños M, Correro-Bermejo A, Soler-Martins PS, de la Torre-Moyano S (2020). Empathy training in health sciences: A systematic review. Nurse Educ Pract.

[64] Kataoka H, Iwase T, Ogawa H (2019). Can communication skills training improve empathy? A six-year longitudinal study of medical students in Japan. Med Teach.

[65] Shapiro J, Youm J, Kheriaty A, Pham T, Chen Y, Clayma R (2019). The human kindness curriculum: An innovative preclinical initiative to highlight kindness and empathy in medicine. Educ Health (Abingdon).

